# 

**Published:** 1895-04

**Authors:** 


					﻿THE
Southern Medical Record.
A Monthly Journal of Medicine and Surgery.
Vol. XXV.
ATLANTA, GA., APRIL, 1895.
No. 4.
EDITORS:
A. W. GRIGGS, M. D.
j. McFadden gaston, m. d.
WILLIS F. WESTMORELAND, M. D/j
LOUIS H. JONES, M. D.
DAN. H. HOWELL, M. D., Business Manager.
WM. S. GOLDSMITH, M. D., Managing Editor.
Entered at the Post-office at Atlanta, Ga., as Second-Class Matter.
The Eoote & Davies Co., Atlanta, Ga.
				

